# Analysis of the precision and sensitivity to change of different approaches to assess cartilage loss by quantitative MRI in a longitudinal multicentre clinical trial in patients with knee osteoarthritis

**DOI:** 10.1186/ar2543

**Published:** 2008-11-05

**Authors:** Jean-Pierre Raynauld, Johanne Martel-Pelletier, François Abram, Marc Dorais, Boulos Haraoui, Denis Choquette, Peter Bias, Karl H Emmert, Stefan Laufer, Jean-Pierre Pelletier

**Affiliations:** 1 Osteoarthritis Research Unit, University of Montreal Hospital Centre, Notre-Dame Hospital, 1560 Sherbrooke Street East, Montreal, Quebec, H2L 4M1, Canada; 2 ArthroVision Inc., 1871 Sherbrooke Street East, Montreal, Quebec, H2K 1B6, Canada; 3 Research Group in Pharmacoepidemiology and Pharmacoeconomics, Research Centre, University of Montreal Hospital Centre, Hôtel-Dieu Hospital, 3850 rue Saint-Urbain, Montreal, Quebec, H2W 1T8, Canada; 4 Clinical Research, Merckle GmbH, chem.-pharm. Fabrik, Graf-Arco-Strasse 3, Postfach 1780 Ulm-Donautal D-89079, Germany; 5 Department of Pharmaceutical Chemistry/Medicinal Chemistry, Eberhard-Karls-University Tübingen, Auf Der Morgenstelle 8, Tübingen D-72074, Germany

## Abstract

**Introduction:**

Cartilage thickness and volume loss measurements using quantitative magnetic resonance imaging (qMRI) are suggested to detect significant cartilage changes over short time intervals. We aimed to compare these two different approaches looking at the global knee and subregions, using data from an osteoarthritis (OA) multicentre randomised clinical trial.

**Methods:**

Three hundred and fifty-five patients with symptomatic knee OA were recruited for a two-year, double-blind, randomised clinical trial evaluating the effect of 200 mg licofelone twice daily and 500 mg naproxen twice daily on cartilage loss, and 301 patients had baseline MRI. MRIs were performed at baseline, 6, 12 and 24 months. Cartilage volume and thickness in the global joint, medial and lateral compartments, and central weight-bearing subregions of the medial and lateral femoral condyles and tibial plateaus were analysed. Data were analysed for the mean value imputed for intent-to-treat (ITT-MVI) and statistical analyses were performed using two-sample Student's t-test.

**Results:**

Cartilage mean thickness loss in the global joint, lateral and medial compartments, as well as in medial compartments stratified according to patients with or without meniscal extrusion, was significantly less in the licofelone compared with the naproxen group at 12 and 24 months. Interestingly, these data were similar to those found when using cartilage volume loss as an outcome. Although greater cartilage volume and mean thickness loss was seen in central weight-bearing subregions of the medial and lateral compartments compared with the whole compartment and also in patients with meniscal lesions/extrusion, suggesting good sensitivity to change, its high standard deviation precluded for the condyles a high statistical power and abrogated statistically significant differences between the treatment groups.

**Conclusions:**

These data demonstrate that both the measurement of cartilage thickness and that of cartilage volume provide the same level of sensitivity to estimate cartilage loss in a clinical trial. However, the potential of gaining statistical power with the use of thickness/volume change in knee subregions as an outcome seems negated by high inter-patient variability. Moreover, there is no superiority in statistical power by selecting patients with meniscal extrusion.

## Introduction

Osteoarthritis (OA) is characterised by a number of structural changes that include the progressive loss over time of articular cartilage from the joint surfaces. Such loss has been evaluated mainly by arthroscopic and histological assessments in pre-clinical studies and with the use of X-rays in clinical studies. These methods have been recognised as having significant limitations, particularly in the assessment and quantification of cartilage loss in observational studies following the evolution of the disease, and in clinical trials with disease modifying OA drugs (DMOADs).

A number of X-ray methods and techniques have been proposed and recommended for use in the assessment of drug effects in such trials [1-3]. Some success has been achieved in various studies exploring the effects of drugs with DMOAD activity by measuring the change in joint space width in the medial compartment of the knee [1,4-8]. The low sensitivity to change of the X-ray method, and the fact that the loss in joint space width in OA could be related to structural changes other than cartilage loss, such as the presence of meniscal extrusion [9], have raised a number of issues regarding the use of X-rays in such trials.

In recent years, the use of magnetic resonance imaging (MRI) for the assessment of musculoskeletal structural changes has expanded to include a number of new technologies that are capable of quantitatively and precisely evaluating cartilage volume/thickness over the joint surface, allowing for three-dimensional (3D) reconstruction mapping of the entire joint cartilage [10,11]. A first-step approach through segmentation provides a set of two-dimensional (2D) contours that, when followed by a second step of a 3D reconstruction of the entire joint, allows cartilage thickness and volume estimation in the whole joint, as well as in the different compartments, topographical areas and subregions [12].

These methods to assess cartilage volume/thickness can be used in cross-sectional or longitudinal studies. In longitudinal studies, quantitative MRI (qMRI) has been found to be capable of precisely and reliably assessing changes in cartilage volume/thickness over time [11-21]. Moreover, studies have demonstrated that in patients with knee OA, cartilage loss is more severe in the weight-bearing areas of the medial femoral condyles and tibial plateaus [12]. The rate of knee cartilage loss in these patients as measured by MRI was also found to be predictive of the subsequent need for an arthroplasty [22].

The most important question, however, is the usefulness of these new investigative technologies in the context of multicentre clinical trials exploring the DMOAD effects of new drugs. A first DMOAD study has just been completed and the data of the 3D evaluation of cartilage volume loss in the major knee compartments over time in the different treatment groups have been reported [23]. The present report extends the findings from this previous one, and explores in depth and compares different methods of assessing cartilage loss over time using qMRI in a DMOAD clinical trial. More specifically, the sensitivity to change of two methods of measurement, mean cartilage thickness and cartilage volume, was assessed in order to gain insight into which method offers greater sensitivity to estimate cartilage loss over time and which method better estimates differences between treatment groups.

We also explored whether the selective evaluation of subregions where the greatest loss of cartilage occurs, i.e. the weight-bearing areas of the condyles and plateaus, offers more sensitivity to assess changes between treatment groups than evaluation of the loss in the entire condyle, plateau or compartment. These questions of assessing the impact of DMOADs on knee structure are obviously of the utmost importance as they raise issues that cannot be answered solely by observational longitudinal studies, which have been the main focus of attention so far.

## Materials and methods

The original study design and protocol have been previously described in detail [23]. In this multicentre, randomised, double-blind study comparing 200 mg licofelone (Merckle GmbH, Ulm, Germany) twice daily with 500 mg naproxen (Ratiopharm GmbH, Ulm, Germany) twice daily in patients with knee OA, subjects were treated for 24 months. Naproxen was chosen as a comparator treatment because it is one of the most commonly prescribed non-steroidal anti-inflammatory drugs (NSAIDs) for the symptomatic treatment of knee OA. MRI was performed at baseline, 6, 12, and 24 months. Intent-to-treat (ITT) analysis was carried out by imputing the missing value to the average change recorded (mean value imputed; ITT-MVI) among patients within their corresponding treatment group at a specific time point (6, 12 and 24 months), provided the patients had at least a baseline MRI. Two methods of cartilage measurement, volume and mean thickness, were used as previously described [10]. All patients provided written informed consent before entering the study, which was conducted in accordance with International Conference on Harmonisation of Technical Requirements for Registration of Pharmaceuticals for Human Use Guidelines for Good Clinical Practice, and was administered by local and central institutional review boards.

Briefly, MRI acquisition allows a 3D image of knee surfaces to be produced. With the 3D surfaces of the femur and tibia, a bone-to-cartilage interface and a cartilage-to-soft tissue interface are generated. To enhance the accuracy of the numerical processing, a specific coordinate system is used to represent each surface, that is a 3D cylindrical coordinate system for the femur and 3D space coordinate system for the tibia [10]. The sampling used for the representation of these surfaces is independent of the acquisition resolution. The choice of the 3D coordinate system allows each point of cartilage measurement to be represented by two axes (x and y) along the cartilage surface, while the third axis (z) is perpendicular. The cartilage thickness is represented by the Euclidean distance (z) between the bone-to-cartilage interface and the cartilage-to-soft tissue interface at each sample. The cartilage volume is derived from the thickness and location of both surfaces at each sample location as previously described [10].

The change in volume and mean thickness over time was obtained by subtracting the follow-up value (volume or mean thickness) from the initial (baseline) value. The percentage of cartilage loss was calculated by dividing the change (volume or mean thickness) by the baseline value, and the cartilage loss over time evaluated for the entire knee (global) and for each of the medial and lateral compartments. It was also calculated for: the subregions of the medial central condyle and tibia (transversal slices) as described previously [12]; the lateral central condyle and tibia (transversal slices); and the medial compartment and medial central condyle and tibia (transversal slices) stratified by the absence or presence of meniscal extrusion.

The extent of meniscal extrusion on the medial or lateral edges of the femorotibial joint space was evaluated for the anterior, middle and posterior horns of the menisci in which 0 = no extrusion, 1 = partial extrusion and 2 = complete extrusion with no contact with the joint space (severe). The percentage of cartilage loss in the medial compartment stratified by the absence (0) or presence (1 or 2) of meniscal extrusion was calculated as previously described [24].

Between treatment groups, variable changes at all time points were assessed using a two-sample Student's t-test. All tests were two-sided, and a p ≤ 0.05 was considered statistically significant. All statistical analyses were performed using SAS software, version 9.1 (SAS Institute Inc, Cary, NC).

## Results

Three hundred and fifty-five patients were enrolled in the study and randomly assigned to receive licofelone or naproxen [23]. Baseline characteristics of this population were previously described [23] (mean age of 60 years, 68% female, average BMI 32 kg/m^2^). Three hundred and one patients, 147 in the licofelone group and 154 in the naproxen group, had a baseline MRI (ITT).

The cartilage loss for the global, lateral and medial compartments is presented in terms of absolute value (Table 1) and percentage (Table 2) for both volume and mean thickness. Both the volume and mean thickness methods produced similar findings of cartilage loss in terms of absolute value and percentage in the global, lateral and medial compartments at each time point. For example, at 24 months, the percentage of cartilage loss in the global compartment with the volume method (licofelone mean ± standard deviation: -5.9 ± 2.2%, naproxen: -7.3 ± 2.5%, p < 0.0001) provided similar findings to those from the mean thickness method (licofelone: -5.4 ± 2.1%, naproxen: -6.7 ± 2.4%, p < 0.0001). This was also the case for the lateral compartment (volume: licofelone: -4.7 ± 3.0%, naproxen: -6.0 ± 3.0%, p = 0.0002; mean thickness: licofelone: -4.3 ± 2.7%, naproxen: -5.7 ± 2.8%, p < 0.0001) and the medial compartment (volume: licofelone: -7.5 ± 3.7%, naproxen: -8.8 ± 4.4%, p = 0.004; mean thickness: licofelone: -6.6 ± 3.3%, naproxen: -8.0 ± 3.9%, p = 0.001).

**Table 1:** Average of absolute value of cartilage loss in the global, lateral and medial compartments at 6, 12, and 24 months of follow-up

	**Volume **(mm^3^)			**Mean thickness **(mm)		
		
	**Licofelone**	**Naproxen**	**p-value**	**Licofelone**	**Naproxen**	**p-value**
**Global**						
6 mo	-309.7 ± 238.9	-357.8 ± 320.3	0.140	-0.043 ± 0.034	-0.051 ± 0.047	0.093
12 mo	-438.7 ± 252.9	-543.3 ± 287.3	0.0009	-0.061 ± 0.034	-0.078 ± 0.040	<0.0001
24 mo	-701.6 ± 331.9	-853.2 ± 299.0	<0.0001	-0.095 ± 0.040	-0.119 ± 0.041	<0.0001
**Lateral**						
6 mo	-130.8 ± 143.7	-159.4 ± 143.4	0.085	-0.038 ± 0.041	-0.046 ± 0.043	0.091
12 mo	-192.1 ± 146.1	-244.3 ± 168.2	0.004	-0.056 ± 0.041	-0.072 ± 0.049	0.002
24 mo	-290.7 ± 184.2	-368.6 ± 161.4	0.0001	-0.080 ± 0.051	-0.105 ± 0.047	<0.0001
**Medial**						
6 mo	-178.9 ± 157.2	-198.3 ± 214.0	0.370	-0.047 ± 0.043	-0.056 ± 0.059	0.136
12 mo	-246.6 ± 185.3	-299.0 ± 182.7	0.014	-0.064 ± 0.050	-0.084 ± 0.049	0.0006
24 mo	-410.9 ± 229.9	-484.6 ± 237.1	0.007	-0.107 ± 0.053	-0.131 ± 0.061	0.0004

Values are presented as mean ± standard deviation. mo = months

**Table 2:** Average of percentage of cartilage loss in the global, lateral and medial compartments at 6, 12 and 24 months of follow-up

	**Volume **(mm^3^)			**Mean thickness **(mm)		
		
	**Licofelone**	**Naproxen**	**p-value**	**Licofelone**	**Naproxen**	**p-value**
**Global**						
6 mo	-2.7 ± 1.9	-3.0 ± 3.2	0.250	-2.4 ± 1.9	-2.8 ± 2.9	0.203
12 mo	-3.9 ± 2.0	-4.8 ± 2.5	0.0002	-3.5 ± 1.9	-4.5 ± 2.3	<0.0001
24 mo	-5.9 ± 2.2	-7.3 ± 2.5	<0.0001	-5.4 ± 2.1	-6.7 ± 2.4	<0.0001
**Lateral**						
6 mo	-2.1 ± 2.3	-2.6 ± 2.6	0.092	-2.0 ± 2.2	-2.4 ± 2.5	0.125
12 mo	-3.3 ± 2.4	-4.3 ± 3.2	0.001	-3.1 ± 2.1	-4.1 ± 2.9	0.0006
24 mo	-4.7 ± 3.0	-6.0 ± 3.0	0.0002	-4.3 ± 2.7	-5.7 ± 2.8	<0.0001
**Medial**						
6 mo	-3.3 ± 2.9	-3.6 ± 5.0	0.560	-2.9 ± 2.7	-3.2 ± 4.3	0.383
12 mo	-4.6 ± 3.2	-5.6 ± 3.5	0.009	-4.0 ± 3.0	-5.1 ± 3.2	0.001
24 mo	-7.5 ± 3.7	-8.8 ± 4.4	0.004	-6.6 ± 3.3	-8.0 ± 3.9	0.001

Values are presented as mean ± standard deviation. mo = months

Table 3 presents the absolute value of cartilage loss in the medial central condyle and tibia (transversal slices) for both the volume and mean thickness measurements. Data showed no difference between the two methods for the medial central tibia at each time point examined: 6, 12 and 24 months. However, a slight difference favouring the mean thickness was observed between the two methods in the medial central condyle at six months only (volume: licofelone: -34.0 ± 53.0, naproxen: -41.3 ± 62.4, p = 0.271; mean thickness: licofelone: -0.040 ± 0.083, naproxen: -0.061 ± 0.102, p = 0.056). Interestingly, for both volume and mean thickness in the condyle there was no statistical significance between treatment groups at 24 months, contrasting with data from the whole medial compartment.

**Table 3:** Average of absolute value of cartilage loss (volume and mean thickness) in the medial central condyle and tibia at 6, 12 and 24 months of follow-up

	**Volume **(mm^3^)			**Mean thickness **(mm)		
		
	**Licofelone**	**Naproxen**	**p-value**	**Licofelone**	**Naproxen**	**p-value**
**Condyle**						
6 mo	-34.0 ± 53.0	-41.3 ± 62.4	0.271	-0.040 ± 0.083	-0.061 ± 0.102	0.056
12 mo	-48.5 ± 52.4	-60.2 ± 52.0	0.053	-0.062 ± 0.085	-0.084 ± 0.082	0.020
24 mo	-91.9 ± 74.9	-102.6 ± 73.9	0.215	-0.130 ± 0.107	-0.141 ± 0.109	0.370
**Tibia**						
6 mo	-41.8 ± 35.9	-40.5 ± 34.5	0.755	-0.099 ± 0.090	-0.097 ± 0.090	0.815
12 mo	-55.8 ± 39.4	-68.7 ± 44.3	0.008	-0.134 ± 0.093	-0.166 ± 0.116	0.010
24 mo	-77.4 ± 45.5	-101.9 ± 55.3	<0.0001	-0.182 ± 0.100	-0.239 ± 0.134	<0.0001

Values are presented as mean ± standard deviation. mo = months

The results of the absolute value of cartilage loss in the lateral central condyle and tibia (transversal slices) for both the volume and mean thickness measurements are described in Table 4. At 24 months, a small trend favouring mean thickness was observed in the central condyle (volume: licofelone: -35.1 ± 60.9, naproxen: -46.3 ± 40.0, p = 0.062; mean thickness: licofelone: -0.047 ± 0.096 naproxen: -0.067 ± 0.072, p = 0.040), whereas at 6 and 12 months both methods provided similar findings. In the tibia, however, data strongly favoured the volume method at 12 months (volume: licofelone: -44.7 ± 32.2, naproxen: -56.1 ± 32.9, p = 0.003; mean thickness: licofelone: -0.124 ± 0.094, naproxen: -0.151 ± 0.095, p = 0.013), and 24 months (volume: licofelone: -71.9 ± 41.1, naproxen: -85.8 ± 41.9 p = 0.004; mean thickness: licofelone: -0.203 ± 0.120, naproxen: -0.232 ± 0.110, p = 0.032). Again, and contrasting with the whole lateral compartment, in the central condyle there was no statistical significance at 12 months between treatment groups.

**Table 4:** Average of absolute value of cartilage loss (volume and mean thickness) in the lateral central condyle and tibia at 6, 12 and 24 months of follow-up

	**Volume **(mm^3^)			**Mean thickness **(mm)		
		
	**Licofelone**	**Naproxen**	**p-value**	**Licofelone**	**Naproxen**	**p-value**
**Condyle**						
6 mo	-21.5 ± 44.2	-27.6 ± 40.7	0.210	-0.031 ± 0.073	-0.040 ± 0.078	0.299
12 mo	-25.5 ± 47.6	-34.6 ± 44.7	0.088	-0.039 ± 0.077	-0.055 ± 0.081	0.078
24 mo	-35.1 ± 60.9	-46.3 ± 40.0	0.062	-0.047 ± 0.096	-0.067 ± 0.072	0.040
**Tibia**						
6 mo	-33.3 ± 33.2	-32.8 ± 39.3	0.899	-0.091 ± 0.098	-0.090 ± 0.110	0.940
12 mo	-44.7 ± 32.2	-56.1 ± 32.9	0.003	-0.124 ± 0.094	-0.151 ± 0.095	0.013
24 mo	-71.9 ± 41.1	-85.8 ± 41.9	0.004	-0.203 ± 0.120	-0.232 ± 0.110	0.032

Values are presented as mean ± standard deviation. mo = months

The analyses stratified by the absence or presence of meniscal extrusion showed similar findings with the volume and mean thickness measurements in the medial compartment (Table 5). In the medial central condyle and tibia (transversal slices) subregions stratified according to the absence or presence of meniscal extrusion, greater cartilage loss was found for the volume (data not shown), although findings were also similar with both methods.

**Table 5:** Average of absolute value of cartilage loss (volume and mean thickness) in the medial compartment stratified by the absence or presence of meniscal extrusion at 6, 12 and 24 months of follow-up

	**Volume **(mm^3^)			**Mean thickness **(mm)		
		
	**Licofelone**	**Naproxen**	**p-value**	**Licofelone**	**Naproxen**	**p-value**
**Absence of meniscal extrusion**						
6 mo	-143.5 ± 126.7	-163.7 ± 211.1	0.399	-0.038 ± 0.036	-0.046 ± 0.057	0.203
12 mo	-198.8 ± 152.1	-253.7 ± 134.6	0.007	-0.051 ± 0.042	-0.072 ± 0.036	0.0003
24 mo	-366.2 ± 192.8	-437.2 ± 174.3	0.006	-0.096 ± 0.049	-0.118 ± 0.044	0.0006
**Presence of meniscal extrusion**						
6 mo	-239.9 ± 184.9	-293.8 ± 194.0	0.171	-0.062 ± 0.049	-0.082 ± 0.057	0.076
12 mo	-329.0 ± 208.5	-423.9 ± 234.9	0.040	-0.085 ± 0.054	-0.117 ± 0.063	0.011
24 mo	-488.0 ± 267.3	-615.2 ± 325.9	0.040	-0.128 ± 0.055	-0.166 ± 0.084	0.013

Values are presented as mean ± standard deviation. mo = months

Comparisons of findings using changes in percentage between volume and mean thickness instead of the absolute value (mm^3^) revealed that 92% of the findings were similar with the two methods.

## Discussion

This in-depth analysis provides interesting new information about the potential and limits of different methods that can be used to analyse data in the assessment of the evolution of cartilage loss and the response to treatment of patients with knee OA in multicentre DMOAD clinical trials exploring and comparing drug effects.

The first important question addressed was whether assessing cartilage loss using the mean thickness approach offers the same level of sensitivity to change as the cartilage volume approach. An initial observation was that the measurement of cartilage volume changes in the global (entire) knee and medial and lateral compartments provided exactly the same level of sensitivity to estimate between-treatment comparative changes in the therapeutic groups over the different time points of the study. The findings were similar when the data were analysed as absolute or relative (percentage) value of cartilage loss. Overall, these results showed that both methods of assessment, that is measuring changes in cartilage volume or mean thickness, offer the same level of sensitivity to evaluate cartilage loss at different times and to estimate the effects of treatment. These findings are in line with the calculation of the correlation coefficients between the changes over time of the two measurements, which are all greater than 0.90 (p < 0.0001, Pearson rho) regardless of the cartilage compartment (global, medial or lateral), the time span (6, 12 or 24 months) or treatment group chosen. This is inherent in the methodology because the cartilage volume and mean thickness computations assess the cartilage in a very similar way.

The findings from the present study provide new information on comparative results of data generated differently, a finding that has not been reported before in the context of such studies. Previous observational longitudinal trials have employed both methods to assess cartilage loss. Although some suggestions have been made of the possible superiority of mean thickness over cartilage volume assessment in such trials, head-to-head data comparison has not been reported [25]. The present study provides a definite answer to that very important question, not only in the context of a longitudinal study, but more importantly in the context of a DMOAD trial involving the assessment of changes over time within a patient treatment group, as well as between study arms. As mentioned above, the cartilage volume calculation being derived from the cartilage thickness, the good correlation between these two methods of measurement was therefore not unexpected.

Another important issue to be addressed in the context of DMOAD trials is whether concentrating on the analysis of cartilage changes in the subregions, where the greatest loss of cartilage is found on the condyles and plateaus, would provide a better chance of finding significant differences between treatment groups and allow these differences to be observed earlier in the course of the trial. The results from longitudinal observational studies are certainly supportive of such an hypothesis [11,12,21,26]. However, in the present study, the selective measurement of the loss of cartilage volume and mean thickness in the central weight-bearing zones of the medial femoral condyles and tibial plateaus demonstrated greater loss, but also greater variability in results. This is well illustrated on the condyles where statistically significant differences between the two drugs were found at 12 months but not at 24 months. These data contrast with those from the analysis of the global knee and the medial and lateral compartments, in which significant differences between treatment groups were observed at both 12 and 24 months. These findings are important, particularly in the context of a DMOAD clinical trial in which the classical primary outcome is based on measuring the loss of cartilage in the medial compartment. Overall, the results of the lateral compartment are interesting, as data from the volume loss show progressive, statistically significant loss of cartilage, although less significant than from the medial compartment. The change in the lateral compartment could eventually be useful as a secondary outcome in DMOAD studies. Data on the lateral central condyle are again supportive of the fact that the analysis of specific regions with greater cartilage loss offers no advantage over the classical approach of analysis by compartment.

Research questions based on an *a priori *hypothesis as to which area of the knee may show the greatest cartilage loss are difficult to answer. OA disease progression variability is such that any chosen knee subregion may not reflect, for a specific patient, the greatest cartilage loss over time. This is not a problem when a broader area of cartilage assessment is chosen, such as the global knee or the compartments.

Previous reports from longitudinal observational studies have stressed the fact that in patients with knee OA the presence of meniscal lesions or extrusion is among the most important risk factors of cartilage loss [24,27]. The results from the present study extend these findings. A greater difference between treatment groups was seen in the absence of meniscal extrusion at 12 and 24 months. Meniscal extrusion could therefore be of importance in the context of clinical trials because it may potentially be used for stratification of patients or as an inclusion/exclusion criterion, which may theoretically impede the evaluation of the potential of a new DMOAD treatment. The analysis of data from patients without medial meniscal extrusion showed a difference in the loss of cartilage in the medial compartment between the two treatment groups with a trend at six months and a significant difference at 12 and 24 months. These findings may indicate that greater sensitivity to change can be achieved by selecting patients without medial meniscal extrusion, although caution should be exercised at this time with regard to these findings. Patients with meniscal extrusion usually lose more cartilage volume/thickness over time, so these findings are in line with those data previously described for the central weight-bearing subregions. Again, higher inter-patient variability could very well explain these differences.

More information gathered from future studies is needed before a final conclusion can be reached. Again, both methods of measurement, that is the percentage of cartilage volume loss and mean thickness loss, were found to provide the same level of accuracy to estimate the differences between the two treatment groups. It is noteworthy that elapsed time is important for the MRI changes because the most significant changes for both measurement techniques were seen after two years. In previous studies [16,28], we demonstrated that if the cartilage changes of a cohort over time are examined, statistically significant results are seen in as early as six months of follow-up. However, the data at six months shown here do not demonstrate statistical significance between the two treatment groups. According to the data presented, it would appear that a window of at least one year is necessary to see a clear separation between the treatment groups. This may be true for the present comparators, that is licofelone compared with naproxen. However, we would not suggest that a one-year study is sufficient for any knee OA clinical trial because comparing treatments that slow down cartilage progression in a similar way may need a longer time span or a much larger number of patients enrolled to show statistical and clinical significance.

## Conclusion

The findings of this study demonstrate that, in the context of DMOAD trials in patients with knee OA, the measurement of cartilage loss estimated as either the change in volume or the change in mean thickness provides the same level of sensitivity to assess cartilage loss over time, as well as differences between treatment groups. Selection of patients with the greatest loss of cartilage based on certain risk factors such as meniscal extrusion, should be carefully considered, because it may not necessarily provide a better chance of identifying differences between treatment groups. The group with the more rapid progression of cartilage loss also presented higher variability between patients, as indicated by the greater standard deviation in this group. Moreover, the findings indicate that in the context of such DMOAD trials, strategies to select high-risk patients and/or to selectively analyse the subregions with greater cartilage loss, do not, in contrast to results from longitudinal observational studies, provide greater sensitivity to change and, therefore, do not provide any advantage over the use of a general patient cohort combined with the analysis of cartilage loss in the global knee or in the compartments.

## Abbreviations

2D: two-dimensional; 3D: three-dimensional; DMOAD: disease modifying osteoarthritis drug; ITT: intent-to-treat; MRI: magnetic resonance imaging; MVI: mean value imputed; NSAID: non-steroidal anti-inflammatory drug; OA: osteoarthritis; qMRI: quantitative magnetic resonance imaging

## Competing interests

JPR is a consultant for ArthroVision Inc. JMP and JPP were consultants for Merckle GmbH and are consultants and shareholders in ArthroLab Inc. and ArthroVision Inc. FA is an employee of ArthroVision Inc. MD is a consultant for ArthroLab Inc. BH and DC received honoraria from ArthroLab Inc. PB and KHE are employees and SL a scientific advisor of Merckle GmbH. SL holds the patent for licofelone.

## Authors' contributions

All authors have read and approved the manuscript and contributed to the study design, data analysis, interpretation of data and drafting and revision of the manuscript. A data review committee (JPP, JMP, PB and SL) analysed the data, and JPR, MD and JMP were responsible for the accuracy of the data.
